# Selenium Treatment Enhanced Clearance of Salmonella in Chicken Macrophages (HD11)

**DOI:** 10.3390/antiox8110532

**Published:** 2019-11-07

**Authors:** Zhexi Liu, Jianwei Huang, Yijuan Nie, Izhar Hyder Qazi, Yutao Cao, Linli Wang, Yue Ai, Guangbin Zhou, Keliang Wu, Hongbing Han

**Affiliations:** 1 Beijing Key Laboratory for Animal Genetic Improvement, College of Animal Science and Technology, China Agricultural University, Beijing 100193, Chinadedicatedforsss@163.com (Y.C.); 15870619927@163.com (L.W.);; 2 National Engineering Laboratory for Animal Breeding, College of Animal Science and Technology, China Agricultural University, Beijing 100193, China; 3 Farm Animal Genetic Resources Exploration and Innovation Key Laboratory of Sichuan Province, College of Animal Science and Technology, Sichuan Agricultural University, Chengdu 611130, China; vetdr_izhar@yahoo.com; 4 Department of Veterinary Anatomy & Histology, Shaheed Benazir Bhutto University of Veterinary and Animal Sciences, Sakrand 67210, Pakistan

**Keywords:** selenium, salmonella, HD11 cells, macrophage, RNA-seq, immune system

## Abstract

As an important micronutrient, selenium (Se) plays many essential roles in immune response and protection against pathogens in humans and animals, but underlying mechanisms of Se-based control of salmonella growth within macrophages remain poorly elucidated. In this study, using RNA-seq analyses, we demonstrate that Se treatment (at an appropriate concentration) can modulate the global transcriptome of chicken macrophages HD11. The bioinformatic analyses (KEGG pathway analysis) revealed that the differentially expressed genes (DEGs) were mainly enriched in retinol and glutathione metabolism, revealing that Se may be associated with retinol and glutathione metabolism. Meanwhile, Se treatment increased the number of salmonella invading the HD11 cells, but reduced the number of salmonella within HD11 cells, suggesting that enhanced clearance of salmonella within HD11 cells was potentially modulated by Se treatment. Furthermore, RNA-seq analyses also revealed that nine genes including *SIVA1*, *FAS*, and *HMOX1* were differentially expressed in HD11 cells infected with salmonella following Se treatment, and GO enrichment analysis showed that these DEGs were mainly enriched in an extrinsic apoptotic signaling pathway. In summary, these results indicate that Se treatment may not only affect retinol and glutathione metabolism in macrophages, but could also inhibit salmonella-induced macrophage apoptosis via an extrinsic apoptotic signaling pathway involving *SIVA1*.

## 1. Introduction

Selenium (Se) was previously regarded as a toxic element [1], but nowadays is well-known as an essential trace element and has been demonstrated to perform many important biological functions with wider implications in many patho-physiological events in humans and animals [2,3]. Due to its broad range of pleiotropic effects [4], Se has been implicated in anti-proliferative [5] and anti-inflammatory functions [6], and has also been shown to perform many essential modulatory roles in the immune systems of both animals and humans [4,7,8]. The cellular biochemistry of Se is also complex and has many moving parts [2,9]. However, it is well established that biological functions of Se are mainly carried out by selenoproteins, which are encoded by 25 genes in humans and 24 genes in mice [10].

The current advancement in our understanding of Se-mediated regulation of redox signaling in immune cells and selenoprotein-related research has opened new windows of Se implication in immuno-biology; however, the mechanisms of many Se-mediated effects in immune function are still poorly understood and require further elucidation [11]. It has been suggested that Se-mediated modulation of the immune system is multifactorial, and specific contextual events determine which systems are affected [7]. In general, adequate levels of Se are required for the activity of virtually all the components of the immune system [7]. For instance, low Se status/level is linked to autoimmune thyroid dysfunction and poorer immune responses. Similarly, higher Se status/Se supplementation has been demonstrated to have anti-viral effects and is linked to improved immune functions [4,12]. Intriguingly, past studies on human and animal models have shown an implication of Se supplementation on proliferation of lymphocytes and increased expression of both interleukin 2 and high-affinity interleukin-2 receptor and T-cell signaling (reviewed in [2,4]). However, it has also been argued that not all kinds of immunological responses are equally impacted by the increasing Se levels [11].

Selenium status/intake is associated with adaptive and innate immune cell functioning [13]. For instance, Se status has been reported to affect the activation of macrophages, and an increased Se intake could protect neutrophils from an oxidative insult. Furthermore, it was reported that dietary Se intake might impact natural killer (NK) cells [13]. At adequate intake levels, Se can promote the proliferation and differentiation of naïve CD4-positive T lymphocytes towards T helper 1 cells, supporting acute cellular immune response [14]. On the contrary, lower Se status has been associated with an increased risk of mortality and poorer immune function. Inadequate Se intake was shown to favor both macrophages towards an M1 phenotype and T cells towards Th2 cells, indicating the potential implication of Se status in shifting the Th1/Th2 balance [11,15]. Selenium, as an antioxidant, modulates host susceptibility or infectious pathogens and could promote the function of adaptive and innate immune cells [6,16] and inhibit the infection of viruses and bacteria [13]. It was reported that Se status can affect immune response after bacterial infection in several animal models. For instance, a compromised innate immune response was observed in Se-deficient mice after infection with *Listeria monocytogenes* [17]. It has been shown that combined supplementation of Se and vitamin E can improve intracellular bacterial killing in blood neutrophils [18]. At the same time, supplementation of humans with 100 μg/day sodium selenite has resulted in the increased gene expression required for protein biosynthesis in isolated blood lymphocytes, pointing to a higher proliferation of lymphocytes at adequate Se intakes [19].

In general, the response curve of Se is U-shaped, as there is a constringe window between inadequate and excessive intakes [1,10]. The beneficial and toxic effects of Se are associated with the dose, chemical form, and bioavailability of Se species [1]. Past evidence also suggests that Se supplementation in already Se-adequate subjects might increase the risk of many fatal diseases such as cancer or type-2 diabetes, leading to increased mortality rates [12,20]. Therefore, understanding the importance of an optimum dose is the critical part for determining Se supplementation, and understanding the relevant metabolic mechanisms can also help us utilize this essential trace element in a rational manner. Hence, robust and adequate recommendations could be made in future.

*Salmonella* Typhimurium is a facultative anaerobic Gram-negative bacterium that can cause food-borne gastroenteritis in humans [21]. It is also an important pathogen of food-borne diseases in several animal species including cattle, sheep, pigs, and chickens. Salmonellosis has brought huge economic losses to the livestock industry. As the first line of defense for immune response, macrophages can recognize pathogens and produce superoxide free radicals and hydrogen peroxides (H_2_O_2_) for defense against invading microbes [22]. Following salmonella invasion in the body, an excessive oxidative stress is observed in macrophages during the process of salmonella clearing. Many studies have identified several host genes that are critical to controlling salmonella infection by operating both innate and acquired immune pathways. Intriguingly, it has been reported that Se levels in immune cells could influence the extent of “oxidative burst” in both phagocytic (e.g., neutrophils and macrophages) and nonphagocytic cells (e.g., T cells). For instance, levels of Se intake have been implicated in influencing the generation of reactive oxygen species (ROS) by immune cells and their downstream microbe-killing effects [11]. However, the mechanistic basis for such Se-medicated effects is still poorly elucidated.

In this study, chicken macrophage cell line HD11 was used to investigate the potential effect of Se on clearing *Salmonella* Pullorum infection. Furthermore, through RNA-seq-based global transcriptome analyses we explored the potential molecular mechanism of a Se-mediated resistance against intracellular salmonella within HD11.

## 2. Materials and Methods

### 2.1. Materials and Cell Culture

The chicken macrophage-like cell line HD11 was maintained in RPMI1640 (Gibco, Carlsbad, CA, USA) supplemented with 10% fetal bovine serum (FBS, Gibco, Carlsbad, CA, USA) in a humidified incubator with 5% CO_2_ at 37 °C, and cells were passaged before 80–90% confluence.

### 2.2. Cytotoxicity Assay

Using 96-well and 60 mm plates, HD11 cells with 100 μL complete medium per hole were seeded at 5 × 10^4^ cells per hole density. Following attachment of cells to the holes, initial PRMI1640 medium was replaced with new PRMI1640 media supplemented with different concentrations (0nM, 10 nM, 100 nM, 500 nM, 1 μM, 2.5 μM, 5 μM, 7.5 μM, 10 μM, and 50 μM) of sodium selenite, and the cells were grown at 37 °C in a humidified atmosphere with 5% CO_2_ for 24 h to evaluate the survival rate using Cell Counting Kit-8 (CCK8, KeyGEN, Nanjing, China).

### 2.3. Bacterial Strain and Growth Conditions

*S.* Pullorum C79-13 was used in this study. The source of *S.* Pullorum was provided by our laboratory. Strain for infection was grown at 37 °C overnight in a shaking incubator at 180 rpm in Luria Bertani (LB) broth supplemented with 50 μg/mL ampicillin. Then, 10 μL of LB medium was placed in a new liquid LB medium and shaken at 200 r/min for 6 h.

### 2.4. Quantification of Intracellular Bacteria Number

Quantification of intracellular bacteria number was used to detect phagocytotic activity of HD11 macrophages. HD11 cells (1 × 10^5^) were inoculated in a 48-hole plate. After adherence to the wall, cells were attacked with bacteria at different multiplicities of infection (MOIs, MOI = 1, MOI = 5, MOI = 10, MOI = 20) for 20 min, and the extracellular bacteria were eliminated with 100 μg/mL gentamicin treatment for 1 h. At the same time, 200 μL/per hole RPMI1640 complete medium with different doses of sodium selenite (100 and 500 nM) were added to the test groups for 1 h, respectively. After 1 h, one aliquot of cells was added into 0.3% triton-x and counted on a coated plate. The other aliquot was replaced with 20 μg/mL gentamicin complete culture medium for 12 h while maintaining the original concentration of sodium selenite. The test results represent the data of three independent samples.

### 2.5. Maintenance of Cell Lines and Infection

HD11 macrophages were maintained in RPMI1640 (Sigma) phenol red-free medium supplemented with 10% FBS. Cell lines were maintained at 37 °C and 5% CO_2_ with regular medium changes. HD11 cells were passaged before 80% confluence. HD11 cells were seeded in 24-well plates. The experiment was divided into four treatments: control group (Con), Se treatment group (SE, salmonella infection group (BC), and Se treatment + salmonella infection group (BS). The control group did not receive any treatment. In the SE group, after 6 h of cell plating, cells were cultured for 12 h with Se medium at a concentration of 100 nM, then collected with Trizol (Ambion, Carlsbad, CA, USA). In the BC group, after 6 h of cell plating, HD11 cells were infected with salmonella (MOI = 10), and after 30 min the cells were collected with Trizol. In the BS group, after 6 h of cell plating, HD11 cells were infected with salmonella (MOI = 10) for 30 min and then replaced with the 100 nM Se medium containing 100 μg/mL gentamicin for 1 h, then the cells were collected with Trizol. Each group comprised three independent replicates.

### 2.6. RNA Sequencing of Cell Transcriptome Libraries

Total RNA was extracted from HD11 cells using an RNA isolation kit (QIAGEN, Hilden, Germany) according to the manufacturer’s protocol. The quality of RNA was analyzed by agarose gel electrophoresis and a spectrophotometer (DS11, ThermoFisher Scientific, Carlsbad, CA, USA). The samples with good RNA quality were selected. The constructed libraries of four differently treated HD11 cells were sequenced using the Illumina Hiseq TM2000 (Illumina, San Diego, CA, USA) sequencing platform.

### 2.7. Quality Control and Differential Expression Analysis

For quality control, raw data were subjected to stringent filtering (i.e., removing low-quality reads and repeated and adaptor sequences), and the obtained clean reads (clean data) were used for further downstream analyses. Subsequent data analyses mainly refer to the transcriptional analyses as previously described [23], which were adopted in our previous study [24]. Briefly, the following software and settings were used to perform these analyses: Initially, Bowtie2 software (http://bowtie-bio.sourceforge.net/bowtie2/index.shtml) was used to index, then Tophat2 (http://ccb.jhu.edu/software/tophat/index.shtml) was used to filter post-sequence alignment to the reference genome (version 3.1). Gene expression levels were calculated by the FPKM (fragments per kilobase of exon per million parts mapping) algorithm using Cufflinks software (http://cole-trapnell-lab.github.io/cufflinks). The formula is described as follows:

$$\mathrm{FPKM}=\frac{10^{9}C}{NL}$$

where C refers to the number of fragments uniquely mapped to the genome, N refers to the number of reads uniquely mapped to all genes, and L refers to the total length of exons in a given gene.

The selection criteria for differentially expressed genes (DEGs) were as follows: Benjamini and Hochberg’s approach was used for controlling the false discovery rate (FDR). As such, *p* values adjusted to <0.05 and the fold change value |log2Ratio| ≥1 were used as a threshold for significant differential expression. Using this cut-off criteria for DEGs, the numbers of up-regulated and down-regulated genes were recorded.

### 2.8. Gene Ontology (GO) and Kyoto Encyclopedia of Genes (KEGG) Pathway Enrichment Analyses

An online gene function analysis website DAVID (https://david.ncifcrf.gov/) was used to perform subsequent Gene Ontology (GO) enrichment analysis of DEGs involved in the main biological functions. Enrichment of DEGs in different biological pathways was analyzed by using the Kyoto encyclopedia of genes (KEGG) database using DAVID bioinformatics resources (https://david.ncifcrf.gov/). Significance was set at a *p*-value < 0.05.

### 2.9. Statistical Analyses

Statistical analysis was conducted by one-way ANOVA and a post-hoc Fisher’s least significant difference (LSD) test using SPSS statistical software (version 20.0, IBM, Almond, NY, USA). Data are expressed as the mean ± standard error. The statistical significance was defined at *p* < 0.05. The test results represent the data of three independent samples.

## 3. Results

### 3.1. Global Transcriptome of HD11 Cells Treated with Selenium

**Figure 1:**
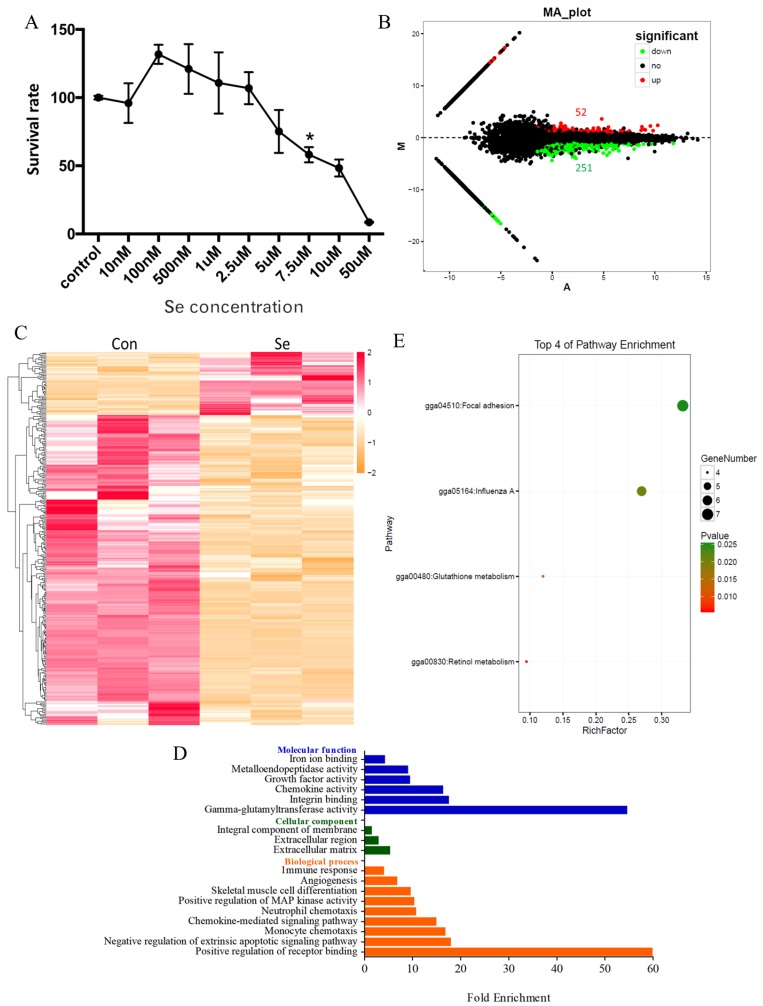
Selenium treatment at an appropriate concentration can promote the survival rate and affect retinol and glutathione metabolism in HD11 cells. (**A**) The survival rate of HD11 cells at different concentrations of Se (sodium selenite) after 24 h. Se at concentrations ranging from 100 nM to 2.5 uM promoted the survival rate of HD11 cells (* indicates the significant difference (*p* < 0.05)). (**B**) The number of DEGs between the control group (Con) and Se-treated group (SE). There were 52 up-regulated genes and 251 down-regulated genes following Se treatment. (**C**) The expression profiles of DEGs between the control group (Con) and Se-treated group (SE). (**D**) The major over-represented GO terms of differentially expressed genes between the control group (Con) and Se-treated group (SE). (**E**) The major over-represented KEGG terms of differentially expressed genes between the control group (Con) and Se-treated group (SE).

A certain amount of Se can promote cell growth and proliferation. However, an excessive Se level has been shown to have cytotoxic effects on cells. Therefore, before investigating the effect of Se on macrophages, we determined the optimal concentration of Se to be used in the subsequent experiments. As shown in Figure 1A, the survival rate of HD11 cells was higher than the control at a low concentration of Se. On the contrary, the survival rate of HD11 was reduced with the increasing Se concentrations, and the survival rate of HD11 cells was 50% at a 7.5-μM concentration of Se (half lethal concentration). The highest survival rate of HD11 cells was observed when cells were treated with 100 nM Se, suggesting that the 100-nM Se concentration was an appropriate dose level for HD11 cells.

To investigate the effect of Se treatment on HD11 macrophage cells, we selected the 100 nM concentration of Se to treat HD11 cells, then analyzed the changes in transcriptome by RNA-seq. The number of DEGs between the control group (Con) and the group treated with Se (SE) is shown in Figure 1B. Briefly, 52 genes were significantly up-regulated and 251 genes were down-regulated. The FPKM of DEGs was clustered in accordance with the test conditions (Figure 1C), demonstrating that addition of Se has a certain modulatory effect on the expression of genes in macrophages. As shown in Table S1, the up-regulated genes included *HMOX1*, *RAX1*, *MMP7*, *GPX1*, *SPP1*, and others, and the down-regulated genes included *CYP3A4*, *CYP3A5*, *UGT2A1*, *GGT7*, *GGT1*, *IL8*, *MMP1*, *MMP9*, *MMP23,* and others (see Table S1).

As shown in Table S2, nine biological process GO terms, three cellular component GO terms, six molecular function GO terms, and fold enrichment were found using *p* < 0.05 to analyze the information regarding GO enrichment terms of DEGs (see Figure 1D and Table S2). The description is as follows: positive regulation of receptor binding (two genes), negative regulation of extrinsic apoptotic signaling pathway (three genes), monocyte chemotaxis (three genes), chemokine-mediated signaling pathway (three genes), neutrophil chemotaxis (three genes), positive regulation of MAP kinase activity (three genes), skeletal muscle cell differentiation (three genes), angiogenesis (seven genes), immune response (five genes) in biological process GO terms. Gamma-glutamyltransferase activity (two genes), integrin-binding (three genes), chemokine activity (three genes), growth factor activity (five genes), metalloendopeptidase activity (eight genes), and iron ion binding (five genes) in molecular function GO terms. Extracellular matrix (five genes), extracellular region (nine genes), integral component of membrane (40 genes) in cellular component GO terms. KEGG pathways in DEGs were analyzed based on the KEGG database. The significant enrichment pathways were obtained, including retinol metabolism (gga00830, four genes), glutathione metabolism (gga00480, four genes), influenza (gga05164, six genes), and focal adhesion (gga04510, seven genes) (see Figure 1E and Table S3), then we picked up the genes associated with retinol metabolism and glutathione metabolism from the global transcriptome in Figure S1, highlighting that Se played an important role in retinol and glutathione metabolism as well as viral infection in macrophages.

### 3.2. The Global Transcriptome of HD11 Cells Infected with Salmonella

**Figure 2:**
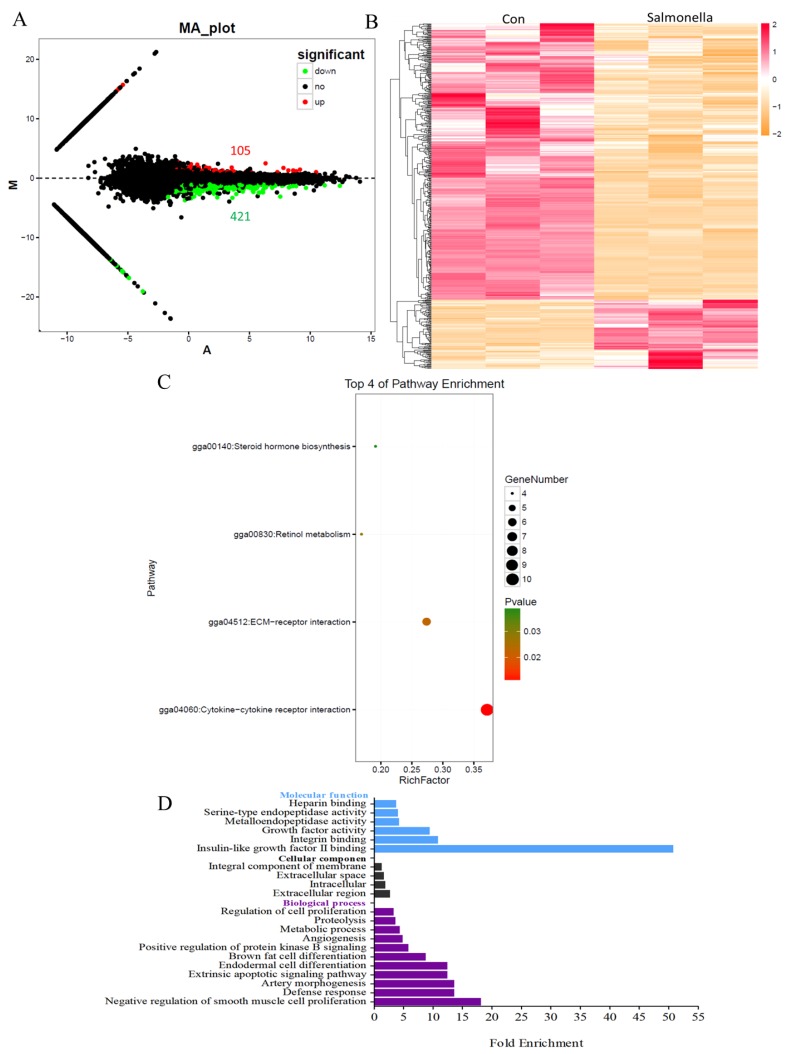
Defense response, extrinsic apoptotic signaling pathway, and metabolic process were affected by salmonella. (**A**) Number of DEGs between the control group (Con) and the group infected with *Salmonella* Pullorum C79-13 (BC), with 105 up-regulated genes and 421 down-regulated genes following salmonella infection. (**B**) Expression profiles of DEGs between the control group (Con) and the group infected with *S.* Pullorum C79-13 (BC). (**C**) Major over-represented GO terms of differentially expressed genes between the control group (Con) and the group infected with *S.* Pullorum C79-13 (BC). (**D**) Top four pathway enrichments of differentially expressed genes between the control group (Con) and the group infected with *S.* Pullorum C79-13 (BC).

To explore the changes in the global transcriptome of macrophages infected with salmonella, HD11 cells were exposed to salmonella at MOI = 10. Subsequent transcriptional analyses were carried out by RNA-seq. In total, 526 DEGs were identified between the control group and the salmonella-infected group, including 104 significantly up-regulated genes and 421 down-regulated genes (Figure 2A and Table S4). As shown in Figure 2B, FPKM of DEGs was clustered in accordance with the test conditions, showing that the gene expression pattern was distinctly different between two groups. The up-regulated genes included *IRG1*, *SIVA1*, *IL8L1*, *IL8*, *HMOX1,* and others, and the down-regulated genes included *FAS*, *G0S2*, *TLR3*, *AvBD3*, *MMP1*, *MMP2*, *MMP9*, *UGT2A1*, *CYP26B1*, and others (Table S4).

The DEGs were analyzed by GO enrichment using the DAVID bioinformatics tool, and the number of genes in each term was counted. As shown in Figure 2C and Table S5, there were 11 biological process GO terms, four cellular component GO terms, and six molecular function GO terms based on *p* < 0.05 and fold enrichment. In the biological processes, the significantly enriched GO terms were as follows: defense response (three genes), extrinsic apoptotic signaling pathway (four genes), metabolic process (five genes), regulation of cell proliferation (seven genes), positive regulation of protein kinase B signaling (five genes), and others. In the cellular component, the enriched GO terms were as follows: extracellular region (15 genes), intracellular (16 genes), extracellular space (20 genes), and integral component of membrane (62 genes). In the molecular function, the enriched GO terms were: insulin-like growth factor II binding (two genes), integrin-binding (three genes), growth factor activity (eight genes), metalloendopeptidase activity (six genes), serine-type endopeptidase activity (six genes), and heparin-binding (five genes). Based on the KEGG pathways database, the significant pathways enriched in DEGs were analyzed. The top four KEGG pathways were sorted by *p*-value and fold enrichment between the control and salmonella-infected groups (Figure 2D). These included steroid hormone biosynthesis (gga00140, four genes), retinol metabolism (gga00830, four genes), ECM-receptor interaction (gga04512, six genes), and cytokine-cytokine receptor interaction (gga04060, 10 genes) (Table S6).

### 3.3. Effect of Se on Clearance of Salmonella within HD11 Cells

**Figure 3:**
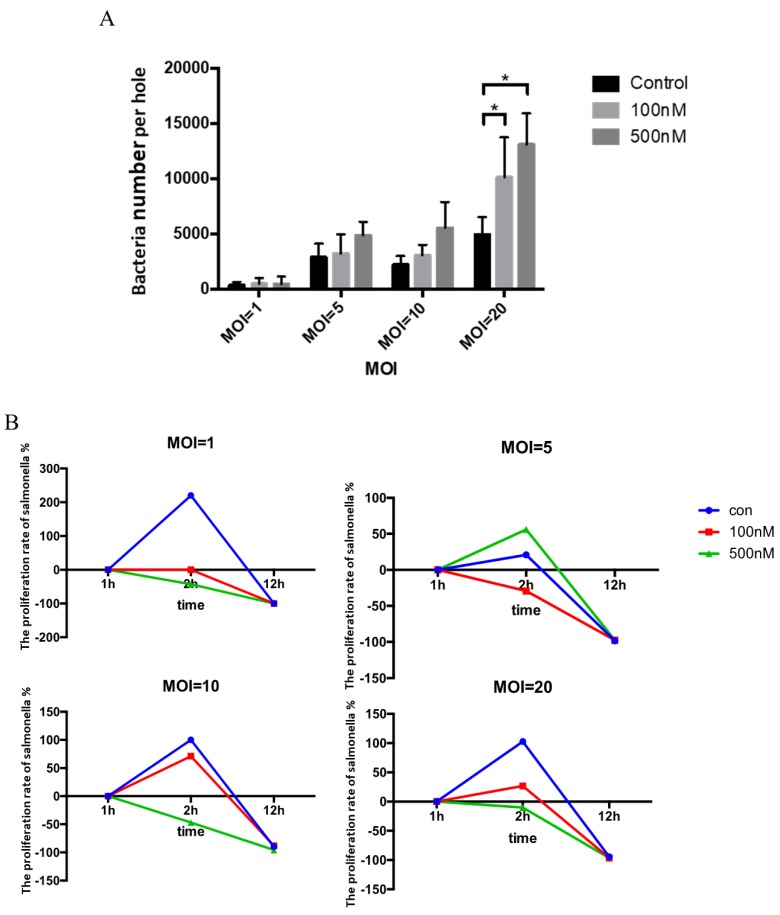
Selenium enhanced the clearance of salmonella within HD11 cells. (**A**) Bacteria number per hole after gentamicin treatment (* indicates a significant difference *(p* < 0.05). The number of bacteria in HD11 cells pre-treated with different concentrations of Se (sodium selenite) showed an increasing trend at different MOI levels. (**B**) Bacteria clearance rate after 0, 2, and 12 h in Se-stimulated HD11 cells. The proliferation rate of salmonella within DH11 cells treated with 100 and 500 nM of Se was decreased substantially at 2 h post-infection at different MOI levels.

It is known that Se can modulate and improve the immune function in humans and animals and can inhibit the invasion of viral and bacterial pathogens. Therefore, in the next experiment we investigated the potential effect of Se treatment on the growth of salmonella within HD11 cells. For this purpose, based on concentration screening experiment, 100 and 500 nM concentrations of Se (sodium selenite) were selected to stimulate HD11 cells followed by salmonella infection at MOI = 1, MOI = 5, MOI = 10, and MOI = 20, respectively. HD11 cells pre-treated with 100 and 500 nM of Se were infected with salmonella for 30 min. Then, the extracellular bacteria were killed by gentamicin exposure (100 ug/mL) for 1 h. At the start point (0 h) of salmonella invasion in HD11 cells, the number of live salmonella within HD11 cells was calculated. As shown in Figure 3A, compared to the control, the number of bacteria (per hole) in HD11 cells pre-treated with different concentrations of Se showed an increasing trend at different MOI levels that was significantly higher compared to the control group at MOI = 20, suggesting that Se can contribute to salmonella invasion into HD11 cells. To further investigate the effect of Se on the salmonella clearance ability of HD11 cells, we analyzed the number of live salmonella within HD11 cells from 2 to 12 h post-infection (PI) at different MOI levels. When compared to the control group, the proliferation rate of salmonella within DH11 cells treated with 100 and 500 nM of Se was substantially decreased at 2 h PI at MOI = 1, MOI = 10, and MOI = 20, respectively, but was comparable at 12 h PI (Figure 3B). However, the proliferation rate of salmonella within DH11 cells treated with 500 nM of Se was higher compared to the control at MOI = 5 (Figure 3B). These data show that Se can inhibit the growth of salmonella within HD11 cells at the early phase of infection. Based on the above results, it is evident that Se pre-treatment increased the degree of salmonella invasion into HD11 cells, but reduced the number of salmonella within HD11 cells, suggesting that Se could potentially promote the ability of macrophages to eliminate a greater number of salmonella during the early phase of infection.

### 3.4. Transcriptome of HD11 Cells Infected with Salmonella Following Se Treatment

**Figure 4:**
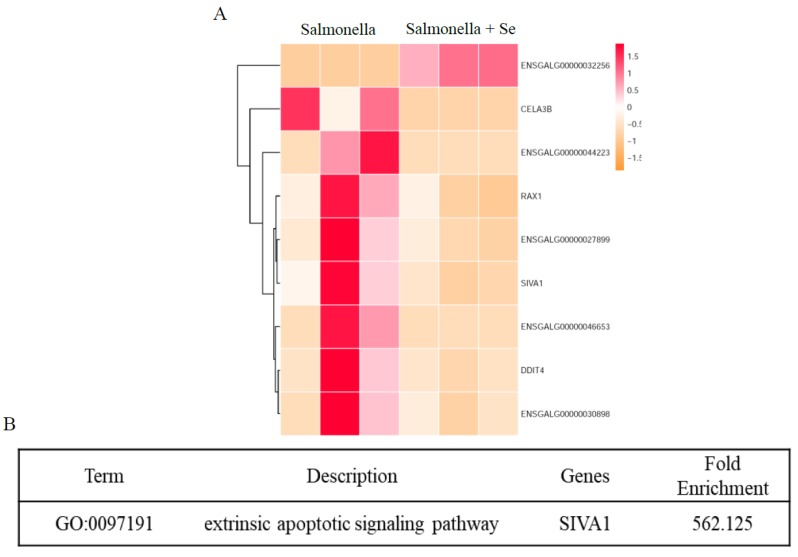
Selenium could inhibit the salmonella-induced macrophage apoptosis through an extrinsic apoptotic signaling pathway via *SIVA1*. (**A**) The expression profiles of DEGs between the group infected with *S.* Pullorum C79-13 (BC: salmonella) and the group of macrophages infected with *S.* Pullorum C79-13 and treated with Se (sodium selenite) (BS: salmonella + Se). (**B**) The major over-represented GO terms of differentially expressed genes between the group infected with *S.* Pullorum C79-13 (BC: salmonella) and the group of macrophages infected with *S.* Pullorum C79-13 and treated with Se (sodium selenite) (BS: salmonella + Se).

To explore the potential basis through which Se treatment improved the salmonella clearance ability of HD11 macrophages using RNA-seq, we carried out transcriptome analysis of HD11 cells infected with salmonella followed by Se exposure. The number of DEGs between the salmonella-infected and the salmonella-infected + Se-treated groups was calculated. The criteria for the selection of DEGs were FDR (*p*-adjust) < 0.05 and a fold change value |log2Ratio| ≥ 1. As shown in Figure 4A, two different trends of gene expression were noted (i.e., one significantly up-regulated and eight down-regulated genes, including *RAX1*, *SIVA1*, *DDIT4*, ENSGALG00000027899, ENSGALG00000046653, ENSGALG00000044223, ENSGALG00000032256, and ENSGALG000000 30898). These genes are involved in basic immune regulation, growth, and development of the organism pathways. Based on the GO database, the enrichment GO terms in DEGs were analyzed by fold enrichment. Our downstream analyses revealed that the extrinsic apoptotic signaling pathway was the most significantly enriched GO term (Figure 4B), implying that Se may control, to some extent, the growth of salmonella within HD11 cells via *SIVA1* gene in extrinsic apoptotic signaling pathway. We then picked up the expression of *SIVA1* from the global transcriptome for two different conditions (C vs BC and BC vs BS), as shown in Figures S2 and S3. These results, somehow, highlight that the expression of *SIVA1* was changed/modulated following Se (sodium selenite) treatment, and intriguingly imply that an appropriate concentration of Se might protect cells against salmonella infection through inhibiting macrophage apoptosis.

## 4. Discussion

Macrophages play an essential role in eliminating pathogens, and represent the first line of defense and mediate a pivotal innate immune response against bacterial infections. Interestingly, macrophages demonstrate remarkable plasticity, and in response to environmental stimulus can change their physiological state and functions [25]. Upon infection, salmonella can invade macrophages and induce cell death or establish an intracellular survival within phagocytic vacuoles [26]. The reactive oxygen species produced by immune cells are usually linked with the phagocyte-mediated killing of microorganisms [11]. Moreover, ROS generated by immune cells in response to invading microbes has been demonstrated to act as the crucial mediators of cellular signaling and communication for several types of phagocytic (i.e., macrophages) and non-phagocytic cells (T cells) in the immune system [11]. It well-established that Se is an essential trace element for humans and animals that has various biological functions, including the protection of cells and tissues from oxidative insult through redox regulation [2]. Although it has been shown that Se could improve innate immunity via modulating macrophage functions [13,27], our understanding of exact underlying molecular mechanisms is still incomplete. However, it has been argued that not all kinds of immune responses are equally influenced by Se levels [11]. The potential mechanistic basis of this caveat is still poorly known, perhaps due to our poor understanding of underlying mechanisms through which Se might modulate the immune response. Therefore, as a logical essential step it is increasingly important to identify the specific cellular signaling pathways and immune functions that are potentially modulated by Se levels [11].

Generally, animal feeds are supplemented with inorganic forms of Se such as, sodium selenite [28]. Considering its potential toxicity and interactions with other minerals, dietary levels of sodium selenite are very cautiously determined [29,30]. In this study, sodium selenite was used as a source of Se treatment, and through concentration screening experiments we determined an appropriate Se dose. HD11 macrophages were exposed to sodium selenite at high and low concentrations, and results revealed that sodium selenite at concentrations of 100 and 500 nM improved the cell survival rate. The rate of HD11 cell death increased with increasing concentrations of sodium selenite, and 2.5 μM was shown to be the critical concentration limit. To our relief, similar effects have been observed in other studies using PC12 cells, NB4 cells, and different malignant glioma cells (U87MG, T98G, A172, U343, U251) [31,32,33]. At the same time, PC12 cells were found to be dead when treated with Se at concentrations higher than 10 μM [14,33], and NB4 cells and glioma cells were found to be dead at 1 and 5 μM of Se treatment. Sodium selenite has also been shown to exert cytotoxic effects on various cancer cells, highlighting its apoptosis-inducing effect [16,25,34]. Some in vivo experiments have demonstrated similar results, where it was shown that, as compared to mice fed a Se-deficient diet, T cell proliferation in the thymus of mice fed a Se-enriched diet was significantly increased, and NK cells were activated [6]. These findings support the fact that Se, at an excessive level, has a toxic effect on cells, and an appropriate concentration of Se can promote cell proliferation.

In this study, using RNA-seq analyses, we investigated the differential gene expression in HD11 cells treated with Se. In total, 303 DEGs were identified that were enriched in four different pathways. For the retinol metabolism, genes included: *CYP3A4*, *CYP3A5*, *UGT2A1*, and *CYP26B1*. It has been reported that Vitamin A has a synergistic effect with Se, as the combined addition of vitamin A and Se is more beneficial to vitamin A metabolism [35]. In vivo studies have demonstrated that the expression of *CYP26B1* is related to vitamin A concentration in a dose-dependent-manner [36]. The significant down-regulation of retinol metabolism-related genes in this study suggests that retinol metabolism in macrophages may be down-regulated following Se treatment. In addition, Se also affects glutathione metabolism in HD11 cells, involving *GPX1*, *GGT7*, and *GGT1*. *GGTs* are involved in the synthesis of glutathione to protect cells from oxidation, and it has been shown that oxidative stress can induce the up-regulation of *GGT* [37]. The *GGT* family modulates crucial redox-sensitive functions, such as anti-oxidant/anti-toxic defense and cellular proliferative/apoptotic balance, suggesting that it plays an important role in tumor progression, invasion, and drug resistance [38,39]. The down-regulation of *GGT1* and *GGT7* in HD11 cells treated with Se indicates that Se alleviates oxidative stress in macrophages. Selenium can up-regulate the expression of *GPX1* (a selenoprotein gene) in humans, mice, and other animals, and a similar finding was obtained in this study. Previously it has been demonstrated that Se can improve the innate immunity of cells and organisms [39]. In our study, the expressions of innate immune response-related genes such as *TNFAIP3*, *IL8*, *MMP1*, *MMP9*, *MMP7*, *MMP23*, and *SPP1* in HD11 cells were modulated by Se treatment. Up-regulated expression of *SPP1* can inhibit apoptosis [40], and, intriguingly, *SPP1* was significantly up-regulated in the Se-treated HD11 cells in the current study, suggesting that Se might inhibit macrophage apoptosis through the increased expression of *SPP1*. *TNFAIP3* is involved in the cellular response to H_2_O_2_ and negatively regulates B cell activation, the CD40 signaling pathway, inflammatory response, and the death domain receptor exogenous apoptosis signaling pathway [41,42,43,44]. Several reports have suggested that *TNFAIP3* also restricts innate immune signaling in response to certain viral infections [45,46,47]. Our results show that *TNFAIP3* in Se-treated macrophages was significantly down-regulated, indicating that the immune functions of macrophages following Se treatment were potentially improved via similar signaling mechanisms.

The peculiar and canonical antioxidant properties of Se are able to produce a protective effect against free radicals and positively contribute to improving the intracellular digestion of phagocyted bacteria. Selenium deficiency reduces the phagocytosis in macrophages in mice [48]. Our results show that the infection rate of HD11 cells treated with Se was significantly higher than that of cells without Se treatment, suggesting that Se can promote phagocytosis of macrophages. However, the proliferation rate of salmonella in Se-treated HD11 cells was lower than that in the cells without Se, indicating that Se (sodium selenite) could inhibit the proliferation of *S.* Pullorum during the early stage of infection.

Interestingly, in salmonella-infected HD11 cells, RNA-seq results showed that 526 DEGs were enriched in retinol metabolism, the extrinsic apoptotic signaling pathway, regulation of cell proliferation, and cytokine-cytokine receptor interactions. The extrinsic apoptotic signaling pathway included *FAS*, *G0S2*, *SIVA1*, and *TLR3* genes. It was reported that *FAS*, a member of the death receptors family, initiated apoptosis by recruiting Fas-associated death domain protein, procaspase-3, and procaspase-8, especially in the immune system [49,50]. Our results showed that the expression of *FAS* in HD11 cells was down-regulated following salmonella infection, indicating that apoptosis of macrophages may be impaired. *Siva1* can induce apoptosis in T cells through a caspase-dependent mitochondrial pathway [51,52]. The expression of *SIVA1* was up-regulated in HD11 cells infected with salmonella, indicating that salmonella may potentially promote macrophage apoptosis via an extrinsic apoptotic signaling pathway involving the *SIVA1* gene.

Moreover, using RNA-seq, we investigated the changes in gene expression in salmonella-infected HD11 cells following Se treatment. Nine differentially expressed genes were found in HD11 cells infected with salmonella then treated with Se (sodium selenite). From these, the expression of *SIVA1*, a pro-apoptotic gene, was down-regulated. Previously it was reported that Siva1 can bind to CD27 and other receptors to induce apoptosis via the caspase mitochondrial pathway in various cell lines [52,53,54]. The expression of *SIVA1* in salmonella-infected HD11 cells treated with Se was down-regulated, while the expression of *SIVA1* in HD11 cells only infected with salmonella was up-regulated, indicating that Se treatment may inhibit salmonella-induced macrophage apoptosis through an extrinsic apoptotic signaling pathway involving *SIVA1*.

## 5. Conclusions

To sum up, we investigated the changes in the global transcriptome of HD11 cells treated with Se (sodium selenite) using RNA-seq, and the results revealed that Se may be associated with retinol and glutathione metabolism, as well as viral infections in macrophages. In addition, our results also showed that Se can potentially promote the HD11 cell phagocytosis, and inhibit the proliferation of salmonella within HD11 cells during the early stage of infection. Furthermore, the potential mechanism was explored by RNA-seq analysis. *SIVA1*, *FAS*, *HMOX1*, and other DEGs were identified in HD11 cells infected with salmonella following Se treatment, and were mainly enriched in an extrinsic apoptotic signaling pathway, as revealed by the GO enrichment analysis. Taken together, our results highlight that Se may not only affect retinol and glutathione metabolism in macrophages, but could also inhibit salmonella-induced macrophage apoptosis via an extrinsic apoptotic signaling pathway involving the *SIVA1* gene. Further focused mechanistic studies are still warranted to improve our understating regarding the Se-mediated modulation of immune functions.

## Supplementary Materials

The following are available online at https://www.mdpi.com/2076-3921/8/11/532/s1: Figure S1. The expression (FPKM) of genes associated with retinol metabolism and glutathione metabolism (Con vs. SE); Figure S2. The expression (FPKM) of *SIVA1* in salmonella-infected HD11 cells (Con vs. BC); Figure S3. The expression (FPKM) of *SIVA1* in HD11 cells infected with salmonella following Se treatment (BC vs. BS); Table S1. The DEGs between the control group (Con) and the group treated with sodium selenite (SE); Table S2. The GO terms between the control group (Con) and the group treated with sodium selenite (SE); Table S3. The KEGG pathway between the control group (Con) and the group treated with sodium selenite (SE); Table S4. The DEGs between the control group (Con) and the group infected with salmonella (BC); Table S5. The GO terms between the control group (Con) and the group infected with salmonella (BC); Table S6. The KEGG pathways between the control group (Con) and the group infected with salmonella (BC).
